# Reliability of Metal 3D Printing with Respect to the Marginal Fit of Fixed Dental Prostheses: A Systematic Review and Meta-Analysis

**DOI:** 10.3390/ma13214781

**Published:** 2020-10-26

**Authors:** Soohyun Bae, Min-Ho Hong, Hyunwoo Lee, Cheong-Hee Lee, Mihee Hong, Jaesik Lee, Du-Hyeong Lee

**Affiliations:** 1 Department of Prosthodontics, School of Dentistry, Kyungpook National University, Daegu 41940, Korea; hyun5601@knu.ac.kr (S.B.); chlee@mail.knu.ac.kr (C.-H.L.); 2 Department of Dental Laboratory Science, College of Health Sciences, Catholic University of Pusan, Busan 46252, Korea; mhhong@cup.ac.kr; 3 Department of Dental Clinic, National Medical Center, Seoul 04564, Korea; surgeonlee@nmc.or.kr; 4 Department of Orthodontics, School of Dentistry, Kyungpook National University, Daegu 41940, Korea; mhhong1208@knu.ac.kr; 5 Department of Pediatric Dentistry, School of Dentistry, Kyungpook National University, Daegu 41940, Korea; leejs@knu.ac.kr; 6 Institute for Translational Research in Dentistry, Kyungpook National University, Daegu 41940, Korea

**Keywords:** metal, 3D printing, reliability, marginal fit, fixed dental prosthesis, systematic review, meta-analysis

## Abstract

Three-dimensional (3D) printing technologies have been widely used to manufacture crowns and frameworks for fixed dental prostheses. This systematic review and meta-analysis aimed to assess the reliability of the marginal fit of 3D-printed cobalt-chromium-based fixed dental prostheses in comparison to conventional casting methods. Articles published until 25 June 2020, reporting the marginal fit of fixed prostheses fabricated with metal 3D printing, were searched using electronic literature databases. After the screening and quality assessment, 21 eligible peer-reviewed articles were selected. Meta-analysis revealed that the marginal gap of the prostheses manufactured using 3D printing was significantly smaller compared to that manufactured using casting methods (standard mean difference (95% CI): −0.92 (−1.45, −0.38); Z = −3.37; *p* = 0.0008). The estimated difference between the single and multi-unit types did not differ significantly (*p* = 0.3573). In the subgroup analysis for the measurement methods, the tendency of marginal discrepancy between the 3D printing and casting groups was significantly different between articles that used direct observation and those that used the silicone replica technique (*p* < 0.001). Metal 3D printing technologies appear reliable as an alternative to casting methods in terms of the fit of the fixed dental prostheses. In order to analyze the factors influencing manufacturing and confirm the results of this review, further controlled laboratory and clinical studies are required.

## 1. Introduction

Three-dimensional (3D) printing is a process wherein a product is manufactured using layering materials from 3D digital data [1,2]. This process enables the fabrication of sophisticated customized products without complicated laboratory manual works, unlike conventional manufacturing methods [3]. Powder bed fusion is a form of a 3D printing technology commonly used for metal 3D printing [4,5]. This technology is also referred to as selective laser sintering (SLS), selective laser melting (SLM), direct metal laser sintering (DMLS), or electron beam melting (EBM) in the literature [6,7]. During the procedure of powder bed fusion, a high-powered laser beam is directed at the layer of metal powder following the design of an object and fuses the powder particles sequentially until the 3D object is completely formed [8]. The main differences between the metal 3D printing technologies are operational parameters and post-treatment processes [9,10].

Cobalt-chromium (Co-Cr) alloys are primarily viable for metal 3D printing in dental prostheses [4]. These alloys demonstrate good tensile strength, elasticity, fatigue resistance, or corrosion resistance [11]. The major constituents of the powder are cobalt and chromium; however, molybdenum, tungsten, silicon, manganese, niobium, cerium, iron, and carbon are also included to improve the properties of the material [9]. Chromium increases corrosion resistance by forming a passivation film; molybdenum influences the grain size and decreases the susceptibility to pitting corrosion; tungsten causes solid solution strengthening with molybdenum; silicon and manganese are inserted to enhance the alloy’s fluidity; and niobium affects the intermetallic phase formation and solution strengthening. Generally, the particles used in the powder bed fusion must be approximately 3–14 µm in size [10], and the products are characterized by the anisotropic γ-phase and ε-phase [12]. Gold and titanium alloys can be processed with the powder bed fusion technology; however, this method is rarely used in dental prostheses owing to the higher cost and mechanical properties [8].

Metal 3D printing technologies have been increasingly used in the field of dentistry for the manufacturing of crowns and frameworks of fixed dental prostheses; however, the accuracy of 3D-printed dental prostheses has not been fully elucidated. Although previous experimental and review trials have compared the accuracy of 3D printing and conventional fabrication methods, the comparisons were limited to individual settings and descriptive analyses [6,7,13]. In the evaluation of the prognosis of fixed prostheses, an acceptable marginal fit is considered a key criterion that influences the clinical success of prostheses [14]. A large marginal discrepancy can influence the cement dissolution and plaque accumulation along the margins, potentially causing secondary caries, inflammatory marginal gingivitis, or prosthesis loosening [15]. The purpose of this systematic review and meta-analysis was to review the reliability of the marginal fit of the Co-Cr based fixed dental prostheses fabricated using metal 3D printing technologies in comparison with conventional casting methods.

## 2. Materials and Methods

### 2.1. Search Strategy

This systematic review was designed as per the “Preferred Reporting Items for Systematic Reviews and Meta-Analyses” (PRISMA) guidelines [16]. The review was processed to answer the primary population, intervention, comparison, and outcome question [17]: in the fabrication of fixed dental prostheses (P), are the 3D printing methods (I) as reliable as the conventional casting method (C) in terms of marginal fit (O)?

The electronic search was performed on 25 June 2020, using the following databases: PubMed, Scopus, Cochrane, and Science Direct, and were limited to articles published in English only. The formulated search strategy included the use of Medical Subject Heading terms and free-text words with Boolean operators (AND or OR): (“metal”[tiab] OR “alloy”[tiab]) AND (“3D printing”[tiab] OR “additive manufacturing”[tiab] OR “SLS”[tiab] OR “DMLS”[tiab] OR “SLM”[tiab]) AND (“accuracy”[tiab] OR “fit”[tiab] OR “adaptation”[tiab]). A direct online search was performed in order to identify additional articles on Google Scholar.

### 2.2. Inclusion and Exclusion Criteria

In the present review, we included original studies that evaluated the marginal fit of the Co-Cr-based complete-coverage prostheses, including single and multiple units. The eligible studies included in vitro, in vivo, and clinical studies, irrespective of the experimental design. The studies were required to have quantitative results of the marginal fit that were recorded as the marginal gap and the absolute marginal discrepancy. The studies that evaluated partial-coverage prostheses, different materials, and other 3D-printed objects, such as dental implants, removable dentures, and artificial bones, were excluded. Narrative reviews, case reports, and studies that did not include a control group with a casting method were excluded as well. The inclusion and exclusion criteria used in this meta-analysis are described in Table 1.

### 2.3. Study Selection and Data Extraction

Two independent reviewers (S.B., and D.-H.L.) screened the studies as per the aforementioned inclusion and exclusion criteria. A calibration exercise with the two reviewers was conducted to increase the reliability of data collection. First, the searched articles were screened as per their titles and abstracts; thereafter, the full text was read after both the reviewers had agreed to the relevance of an article. Disagreements between the reviewers were resolved via discussion. The Cohen kappa coefficient was calculated to determine the final agreement between the reviewers. Subsequently, the characteristics of the eligible studies were extracted from the studies and tabulated in a spreadsheet (Excel; Microsoft, Redmond, WA, USA). The following details were recorded: name of the first author, year of publication, 3D printing device, material, abutment type, prosthesis type, cementation space between the prosthesis and abutment, measurement parameter, measurement method for the marginal gap, sample size, and the value for the marginal fit. While collecting the marginal fit data, the absolute marginal discrepancy (the distance between the most external point of the prosthesis margin and the finish line of the abutment) was first determined as the outcome parameter. When the value was not provided, the marginal gap (the perpendicular measurement from the internal surface of the crown to the margin of the abutment) was recorded.

### 2.4. Quality Assessment

The quality of the included studies was assessed using the Quality Assessment Tool for Diagnostic Accuracy Studies-2 (QUADAS-2) [18]. The four domains for risk of bias assessment were as follows: patient selection, index test, reference standard, and flow and timing. When at least one domain was rated as high risk, the study was considered to have a high risk of bias in its overall judgment. When more than two domains were scored as unclear, the study was regarded as having an unclear bias risk. The traffic-light plots of the specific domain judgments for each study were drawn within each bias domain using the ROBVIS package for R (R software v.3.6.0; R Foundation for Statistical Computing Platform, Vienna, Austria) [19].

### 2.5. Data Analyses

The standard mean difference (SMD) between the prostheses fabricated using 3D printing and those fabricated using casting methods in each study was calculated with the following equation:

$$\mathrm{SMD}\;=\;\frac{\mathrm{Difference}\;\mathrm{in}\;\mathrm{the}\;\mathrm{mean}\;\mathrm{values}\;\mathrm{of}\;\mathrm{the}\;\mathrm{groups}}{\mathrm{Standard}\;\mathrm{deviation}\;\mathrm{of}\;\mathrm{the}\;\mathrm{measurements}}$$

A value of 0 for the SMD indicated that the two fabrication methods produced the prostheses with the same marginal accuracy.

The heterogeneity among the studies was evaluated using the I^2^ statistic, τ^2^, and *p* value (α = 0.05; I^2^ > 50% was considered to indicate heterogeneity). Based on the resulting heterogeneity, a fixed or random effects model was selected for the meta-analyses of the SMD data. The overall estimate of the SMDs was computed with the inverse variance weighted method, and the effect size estimates were adjusted as per the Hedges method [20]. Subgroup analyses of the SMDs were performed for the different types of prostheses (single and multi-unit) and the measurement methods for the marginal fit (direct observation and silicone replica technique). Publication bias was first assessed by inspecting the asymmetry of the meta-analysis results in a funnel plot. Egger’s linear regression test of the funnel plot was then contacted to calculate the statistical value of publication bias. All the meta-analyses were performed using the Meta package for R (R software v.3.6.0; R Foundation for Statistical Computing Platform, Vienna, Austria).

## 3. Results

### 3.1. Search Results

**Figure 1:**
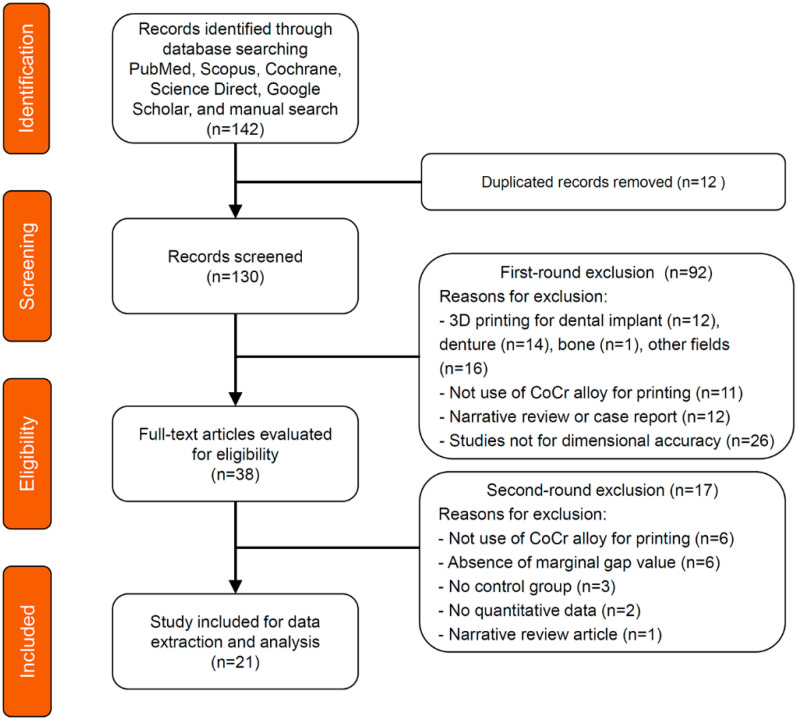
PRISMA flow diagram of the literature search.

The database and manual search initially identified 142 articles. After excluding 12 duplicate articles, 130 articles were screened by reviewing their titles and abstracts. After the exclusion of 91 irrelevant articles, 38 studies were then evaluated for further eligibility. With full-text reading, 17 articles were further excluded as per the inclusion and exclusion criteria; thus, finally, 21 articles were included in the meta-analysis [21,22,23,24,25,26,27,28,29,30,31,32,33,34,35,36,37,38,39,40,41]. The strength of the inter-reviewer agreement for the whole screening process was moderate (κ = 0.463, *p* = 0.010). The search results are described in the PRISMA flow diagram (Figure 1).

### 3.2. Characteristics of the Included Studies

The characteristics of the 21 included studies are summarized in Table 2. In most of the articles, the fit accuracy was investigated on tooth-like abutments; however, there were three studies that used implant abutments. Sixteen studies investigated the fit of single crowns, and five evaluated the fit of a multi-unit fixed dental prosthesis. The mean cement space setting was 35.5 µm (range: 20–70 µm) in 16 studies, excluding the studies that did not provide the set value. In the measurement parameter, two studies showed both the absolute marginal discrepancy and marginal gap values; the other studies showed the marginal gap value alone. In terms of the measurement method for the marginal fit, 11 studies used the silicone replica technique, seven used direction observation, two used mechanical sectioning, and one used a computer-aided digital technique. The sample size in each group was about 10–20 in most studies, except one study that used 110 specimens.

### 3.3. Quality Assessment and Applicability Concerns

**Figure 2:**
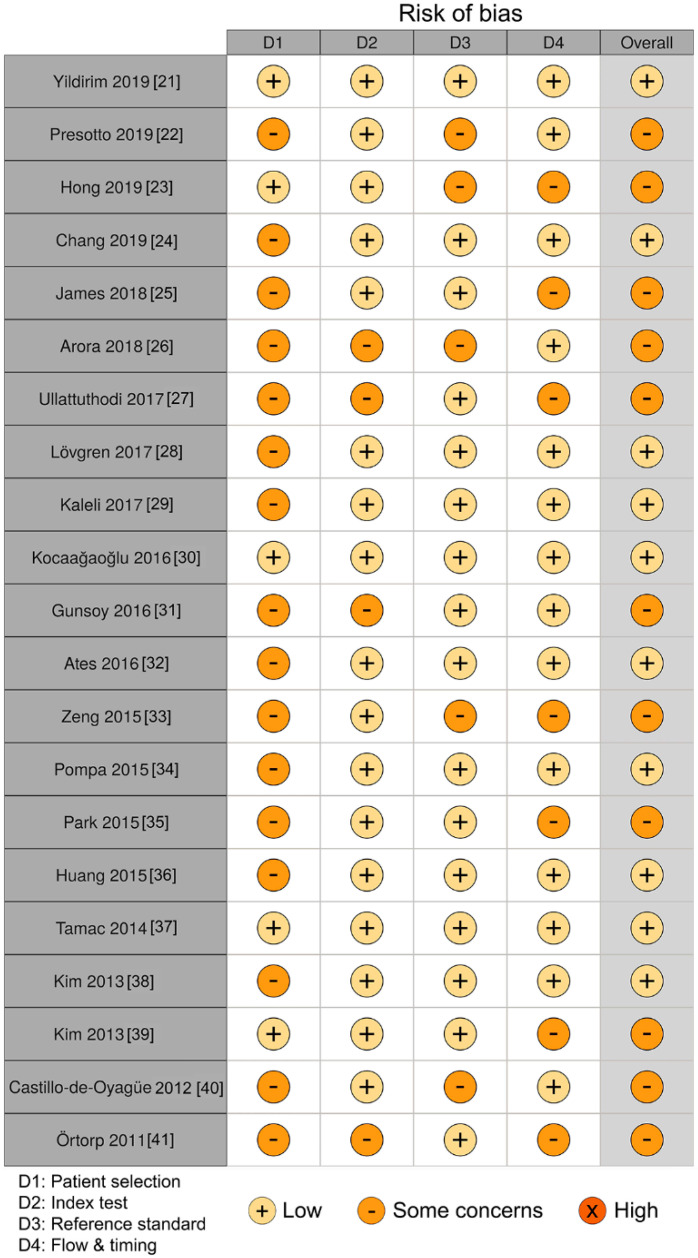
Quality assessment results according to the Quality Assessment Tool for Diagnostic Accuracy Studies-2 (QUADAS-2).

Figure 2 shows the results of the quality assessment using QUADAS-2. Of the 21 articles, 10 exhibited a low risk of bias [21,24,28,29,30,32,34,36,37,38], and 11 articles showed some level of unclear risk of bias [22,23,25,26,27,31,33,35,39,40,41]. All the included studies had a clearly stated purpose and included a control group for the evaluation of the marginal fit. There was major bias in patient selection due to the high number of studies that did not clearly justify the sample size calculation and random sampling. Randomization was not considered necessary because both 3D printing and casting methods were applied to identical specimens, preventing an increase in selection bias. With respect to the index and reference test domains, most studies provided adequate detailed information about the fabrication procedures, parameters, and devices. However, several studies did not explicitly provide the product name of the alloy [26,27,31,32,41] and the setting of cementation space [22,23,26,33,40]. With respect to the flow and timing domain, all the studies clearly reported the measurement methods, positions, and the number of measurement points per specimen. Strategies for achieving measurement reproducibility [24,26,29,30,32,38,40] and blinding tests [22,36,37,40] were provided in several studies to mitigate bias opportunities.

### 3.4. Meta-Analysis

**Figure 3:**
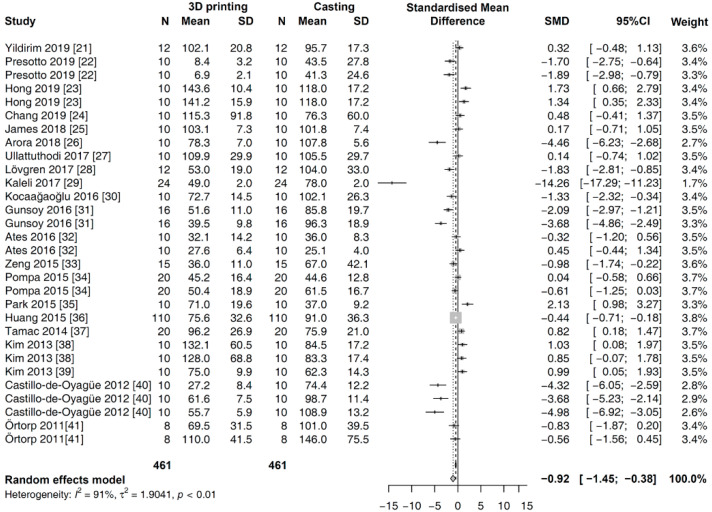
Global meta-analysis on the marginal gap of prostheses fabricated with 3D printing versus casting methods.

In the global analysis, a random effects model was applied to analyze the outcomes of all the studies, considering the heterogeneity among them. The overall SMDs of the marginal gap for two fabrication methods are shown in Figure 3. Pooled analysis revealed that the value of the marginal gap was significantly smaller in the prostheses fabricated with 3D printing methods rather than those made using the casting methods [SMD (95% CI): −0.92 (−1.45, −0.38); Z = −3.37; *p* = 0.0008].

**Figure 4:**
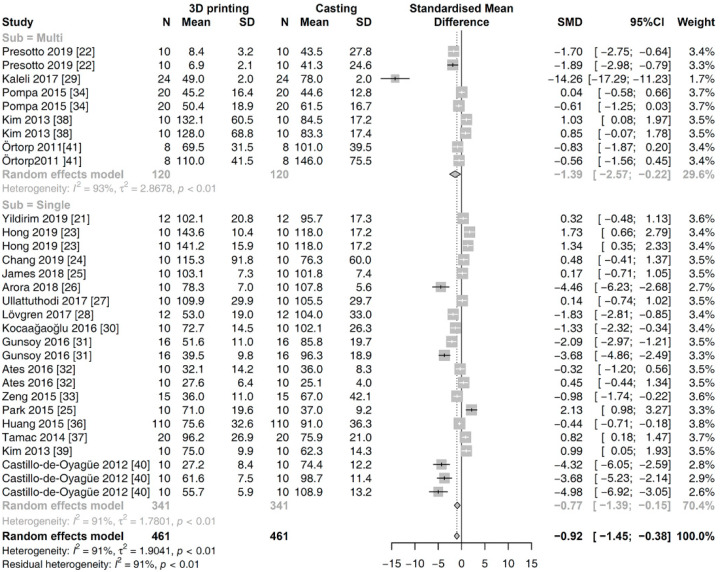
Subgroup meta-analysis for the different prosthesis types on the marginal gap between 3D printing and casting methods.

The first subgroup analysis was performed for different types of prosthesis. There was no significant difference noted in the estimated SMD between single and multi-unit types (*p* = 0.3573). Within the subgroups, the results in the random effects model are as follows (Figure 4): single type [SMD (95% CI): −0.7700 (−1.3871, −0.1529); Z = −2.45; *p* = 0.0145] and multi-unit type [SMD (95% CI): −1.3938 (−2.5697, −0.2178); Z = −2.32; *p* = 0.0202].

**Figure 5:**
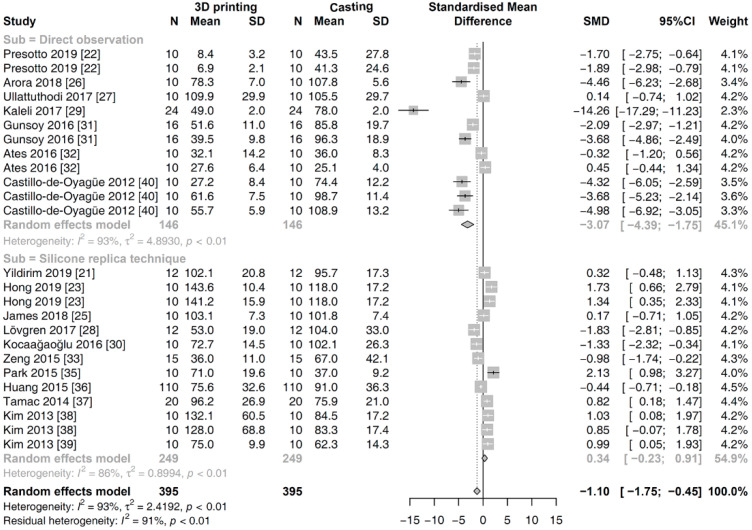
Subgroup meta-analysis for the different measurement methods on the marginal gap between 3D printing and casting methods.

The second subgroup analysis was conducted for the different measurement methods of the marginal fit. There was a significant difference between the subgroups of the direct observation and the silicone replica technique (*p* < 0.001). Within the subgroups, results in the random effects model yielded the following data (Figure 5): direct observation [SMD (95% CI): −3.0735 (−4.3947, −1.7523); Z = −4.56; *p* < 0.001] and the silicone replica technique [SMD (95% CI): 0.3379 (−0.2331, −0.9088); Z = 1.16; *p* = 0.2461].

**Figure 6:**
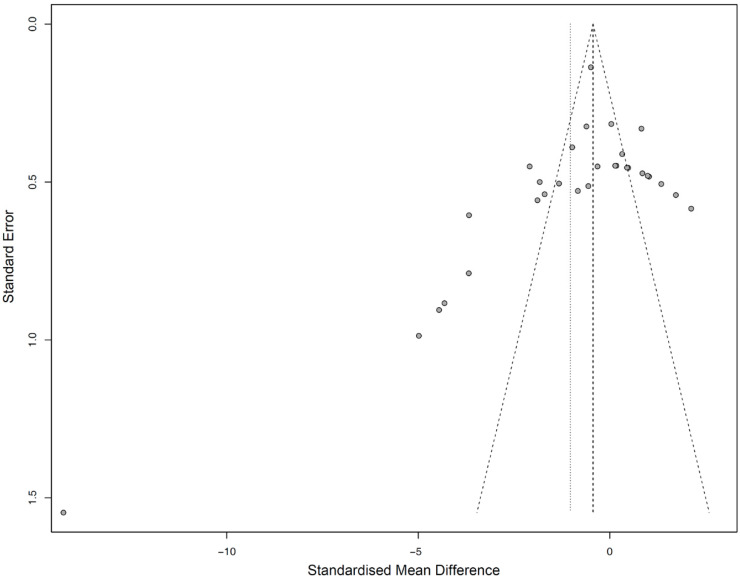
Funnel plot showing publication bias assessment.

Funnel plotting and Egger’s regression test showed a moderate risk of publication bias for both global analyses (*p* = 0.0528) (Figure 6). Accordingly, a random effects model was used to evaluate the SMD results.

## 4. Discussion

This review is aimed at assessing the marginal fit of 3D-printed Co-Cr fixed dental prostheses based on previous original articles. Studies that discussed both 3D printing and conventional casting were systematically selected for a comparative analysis, and statistical meta-analyses were then performed. The results from this review reveal that the marginal gap values of prostheses manufactured with 3D printing were significantly smaller than those of prostheses fabricated using casting methods. Thus, 3D printing technology appears to be capable of providing results comparable or even better than those achieved using prostheses utilizing established fabrication techniques in terms of the marginal accuracy; thus, 3D printing technology can be used as an alternative manufacturing modality. The present results correspond well with earlier narrative reviews wherein the marginal fit of metal frameworks fabricated using 3D printing was within the clinically accepted range [6,7]. To our knowledge, this is the first meta-analysis to compare the 3D printing methods with conventional methods for evaluating the fit of fixed dental prostheses.

The subgroup meta-analysis on the different types of prosthesis presented no significant difference in terms of the estimated SMD between single crowns and multi-unit prostheses. Thus, the 3D-printed prostheses had a better marginal fit than the prostheses fabricated using casting methods, irrespective of the size. Meanwhile, in the subgroup meta-analysis on the measurement methods, the tendency of marginal discrepancy between the 3D printing and casting groups was significantly different between articles that used the direct observation and the silicone replica technique. In the articles with the direct observation, large differences in the misfit were found between the 3D printing and casting groups (SMD = −3.0735, *p* < 0.001). However, within the comparison of the articles with the silicone replica technique, the difference in the misfit between the 3D printing and casting groups was non-significant (SMD = 0.3379, *p* = 0.2461). This finding might be related to the measurement procedure and reliability in the testing methods of the studies [42]. The silicone replica technique is the most commonly used nondestructive method that is applicable in the clinical setting; however, its major drawback is its susceptibility to error [43]. The thin silicone layer in the margin area can be torn or distorted during silicone replica formation. The sectioning of the silicone replica could also be problematic because of human error when the cutting position is inconsistent or the cutting is conducted in oblique planes. When the section is not standardized, the measurements are unreliable [44]. Specific sectioning strategies, such as the use of fiducial markers or cutting devices, have been suggested in several studies to help reduce the possibility of sectioning error [26,29,32,38,40]. Reading error is another issue among observers during the manual selection of measurement points to measure the marginal gap in the acquired images [45]. Thus, suitable standardized procedures are necessary to increase the reliability and reproducibility of the outcomes of individual experiment studies and minimize the bias risk in the meta-analysis performed on the studies.

The fabrication of prostheses via 3D printing begins with the process of digitizing the surface of the prepared abutments with laboratory-based or intraoral optical scanners [46,47]. The scanned image is transferred to a computer design software where the prosthesis is designed. Thereafter, the design is virtually sliced and placed with support structures within the build platform in the computer manufacturing software. In the printing phase, the final prosthesis is manufactured using a stepwise metal powder supply and a laser fusion process. Finally, heat treatment is performed to relieve the internal stress caused by thermal gradients during the whole 3D printing process [48]. The final misfit of 3D-printed prostheses represents the summation of the errors involved in all the steps from the image acquisition to the post-treatment process. Compared to conventional methods, although the digital workflow allows the elimination of inherent inaccuracies related to laboratory manual works, different devices and parameters can highly influence the dimensional accuracy of outcome products [9]. The quality of optical scanners and scan protocols also affect the accuracy of digitization. Technical parameters defined in printing, such as energy source, energy power, laser beam absorption/reflection coefficients, chamber condition, build orientation, layer thickness, and support generation, are all important [10]. The diversity in the manufacturing parameters makes it difficult to make assertive conclusions in the review. Thus, a detailed protocol that discloses all the manufacturing parameters of 3D printing process should be provided to analyze the reliability of a specific 3D printing method, because these factors are commonly interrelated and determine the product quality.

Powder bed fusion technologies use a high-power laser that selectively melts the pre-alloyed powders, following the design of the product, and then partially melts the previous powder layer [2]. The consecutive melting and solidifying of material powders enables interlayer fusion for the completion of the product [8]. Powders for the powder bed fusion are typically produced using gas atomization processes [49]. Spherical particles with a smooth surface and Gaussian size distribution are especially recommended because the spherical shape increases the powder flowability, resulting in higher uniformity and density of powder beds [50]. As the laser beam is shone over the powders, solidification structures such as cells or dendrites are formed in fine and anisotropic microstructures due to the high scanning speeds and small melt pools [51]. Altogether, the particle morphology, particle size distribution, and voids between the powders affect the absorption, reflection, and penetration of the laser radiation [52,53,54]. Consequently, the characteristics of the powder are related to the shape of melt pools and lattice structure. Because the surface roughness and subsequent surface treatment could influence the adaptation of the printed fixed dental prostheses [55,56], it may be necessary to appropriately select powders for the fabrication of dental prostheses.

Close adaptation between mechanical components of implant is essential to minimize biologic and prosthetic complications. The micro gap at the implant–abutment connection is also an important factor that could cause peri-implantitis, and the relationship between the implant–abutment connection and bacterial leakage has been reported in a review [57]. Further meta-analysis on the micro gap and resultant complications might be recommended. In the present review, a meta-analysis on the effects of scanning, printing, and post-treatment conditions could not be performed because the related information was not sufficiently described in the included studies. Most studies reporting on the accuracy of 3D-printed metal frameworks were in vitro studies that involved a bias risk in the justification of the sample size and the random allocation of specimens. We could identify few eligible clinical studies. Therefore, randomized controlled clinical trials will be needed to strengthen the power of review articles. Moreover, considering the rapid development in the fields of technology and material, further original studies with newly developed devices and materials need to be continuously pursued to establish knowledge as per the present times.

## 5. Conclusions

Within the limitations of this systematic review and meta-analysis, the following conclusions were drawn:

1. Metal 3D printing technologies are reliable for fabricating Co-Cr-based fixed dental prostheses with an accurate marginal fit, as compared to conventional casting methods.
2. The difference in methodology for evaluating the marginal fit could influence the results in comparative studies.
3. Further controlled laboratory and clinical studies with a detailed protocol that discloses all the manufacturing parameters are needed to statistically analyze the factors affecting the accuracy of 3D-printed prostheses.

## Figures and Tables

**Table 1:** Inclusion and exclusion criteria used in the meta-analysis.

Inclusion Criteria	Exclusion Criteria
Study Design	Study Design
In vitro study	Case report
In vivo study	Animal studies
Clinical trial	Narrative review
Comparative study	Only charts and questionnaires
Evaluation study	No control group
**Contents**	**Contents**
Assessing marginal fit	Assessing only the whole or internal fit
Complete-coverage prostheses	Direct restorations and partial-coverage prostheses
3D printing using cobalt-chromium alloy	3D printing of dental implants, removable dentures, and artificial bones 3D printing using titanium and gold alloys

**Table 2:** Characteristics of the included studies (n = 21).

	3D Printing			Prosthesis		Measurement		
Study	Device	Material	Ab	Type	Space	Gap	Way	N
Chang 2019 [24]	EOSINT M270, EOS	Wirobond C+, Bego	T	S	30	MG	CD	10
Yildirim 2019 [21]	EOSINT M270, EOS	EOS CoCr SP2, EOS	I	S	25	MG	SR	12
James 2018 [25]	EOSINT M270, EOS	EOS CoCr SP2, EOS	T	S	25	MG	SR	10
Ullattuthodi 2017 [27]	EOSINT M270, EOS	N/P	T	S	50	MG	DO	10
Kocaağaoğlu 2016 [30]	EOSINT M270, EOS	EOS CoCr SP2, EOS	T	S	30	MG	SR	10
Park 2015 [35]	EOSINT M270, EOS	EOS CoCr SP2, EOS	T	S	25	MG	SR	10
Tamac 2014 [37]	EOSINT M270, EOS	EOS CoCr SP2, EOS	T	S	30	MG	SR	20
Kim 2013 [39]	EOSINT M270, EOS	EOS CoCr SP2, EOS	T	S	30	MG	SR	10
Kaleli 2017 [29]	EOSINT M270, EOS	Keramit N/P-S, Nobil Metal	T	M	20	MG	DO	24
Kim 2013 [38]	EOSINT M270, EOS	EOS CoCr SP2, EOS	T	M	30	MG, AMD	SR	10
Hong 2019 [23]	M1, CL	Remanium Star CL, CL	I	S	N/P	MG, AMD	SR	20
Gunsoy 2016 [31]	M1, CL	N/P	T	S	50	MG	DO	16
Lövgren 2017 [28]	Mlab cusing, CL	N/P	T	S	50	MG	SR	12
Presotto 2019 [22]	Mlab cusing, CL	Remanium Star CL, CL	I	M	N/P	MG	DO	10
Ates 2016 [32]	N/P, CL	N/P, Dentaurum	T	S	30	MG	DO	10
Huang 2015 [36]	Medifacturing System, Bego	Wirobond C+, Bego	T	S	70	MG	SR	110
Zeng 2015 [33]	Medifacturing System, Bego	Wirobond C+, Bego	T	S	N/P	MG	SR	15
Arora 2018 [26]	Pro X, 100DP	N/P	T	S	N/P	MG	DO	10
Pompa 2015 [34]	N/P, DeguDent	Starloy LS, DeguDent	T	M	20	MG	MS	20
Castillo-de-Oyagüe 2012 [40]	PM100 Dental, Phenix System	ST2724G, Sint-Tech	T	S	N/P	MG	DO	10
Örtorp 2011 [41]	N/P, Biomain AB	N/P	T	M	50	MG	MS	8

Ab—abutment; CL—concept laser; I—implant; T—tooth; S—single; M—multi; MG—marginal gap; AMD—absolute marginal discrepancy; SR—silicone replica technique; DO—direct observation; MS—mechanical sectioning; CD—computer-aided digital technique; N/P—not provided; N— number of specimen.
